# Burden and determinants of hyperglycaemia and dyslipidaemia in Zimbabwean adults living with HIV on dolutegravir-based first-line antiretroviral therapy: A cross-sectional study

**DOI:** 10.1371/journal.pone.0359196

**Published:** 2026-10-07

**Authors:** Ratidzo Chirimo, Danai Tavonga Zhou, Tinashe Mudzviti, Ian Machingura Ruredzo, Justen Manasa, Charles Chiedza Maponga, Zvenyika Alfred Reginald Gomo, Exnevia Gomo, Gene D. Morse

**Affiliations:** 1 Faculty of Medicine and Health Sciences, University of Zimbabwe, Harare, Zimbabwe; 2 School of Pharmacy, University of the Western Cape, Cape Town, South Africa; 3 Newlands Clinic, Harare, Zimbabwe; 4 Centre for Integrated Global Biomedical Sciences, School of Pharmacy and Pharmaceutical Sciences, University at Buffalo, State University of New York, Buffalo, New York, United States of America; São Paulo State University Institute of Biosciences Languages and Exact Sciences: Universidade Estadual Paulista Julio de Mesquita Filho Instituto de Biociencias Letras e Ciencias Exatas, BRAZIL

## Abstract

**Background:**

Dolutegravir (DTG)-based antiretroviral therapy (ART) is now the preferred first-line regimen for people living with HIV (PLWH) globally, including in Zimbabwe, which has an estimated adult HIV prevalence of 12.9%. While DTG offers a high genetic barrier to resistance and favourable tolerability, it has been linked to metabolic abnormalities — including abdominal obesity, hypertension, dyslipidaemia, and impaired glucose regulation — that cluster together and increase the risk of cardiovascular disease and type 2 diabetes. Data on this burden remain limited in Zimbabwe. We conducted an exploratory, hypothesis-generating cross-sectional study to estimate the burden of hyperglycaemia and dyslipidaemia, and to identify their correlates, among PLWH on first-line DTG-based ART.

**Objective:**

This study aimed to determine the burden and correlates of hyperglycaemia and dyslipidaemia in Zimbabwean adults living with HIV on first-line DTG-based ART.

**Methods:**

A cross-sectional study was conducted from 20 January 2025–13 February 2025. The study participants were 171 PLWH receiving first-line DTG-based treatment at Parirenyatwa Group of Hospitals, in Harare, Zimbabwe. A structured questionnaire was administered to collect demographics, lifestyle information, clinical parameters and anthropometric indices. We calculated body mass index (BMI). Blood pressure measurements were taken with the participant in a resting position. Blood samples were collected and we measured HbA1c, glucose, total cholesterol (TC), HDL cholesterol (HDL-C), LDL cholesterol (LDL-C).

**Results:**

Of the 171 participants, 67.8% were female, with a median age of 42.1 years (IQR 32.4–50.2). The majority had at least secondary education, and few were smokers (n = 9), while 23% reported alcohol use and 62.2% reported no regular exercise. Over half (57.9%) were overweight or obese, and 39.8% were hypertensive. The prevalence of dyslipidaemia and hyperglycaemia was 74.3% and 13.4%, respectively, with insulin resistance present in 56.1%. Non-ideal HDL-C was the most common lipid abnormality (50.5%), followed by raised LDL-C (33.3%), raised triglycerides (16%), and hypercholesterolaemia (10.3%). On multivariate analysis, increasing age was independently associated with hyperglycaemia (AOR 1.08, 95% CI 1.03–1.13, p = 0.002), while elevated blood pressure was independently associated with dyslipidaemia (AOR 2.64, 95% CI 1.03–6.78, p = 0.043).

**Conclusion:**

Dyslipidaemia and hyperglycaemia are highly prevalent among Zimbabwean adults on DTG-based ART, with elevated blood pressure and increasing age as key independent correlates. As this population ages on long-term ART, routine, integrated screening of BMI, blood pressure, glucose, and lipid profile is essential to mitigate cardiometabolic risk and sustain the success of DTG-based ART in Zimbabwe.

## Introduction

It is estimated that 38 million people in the world are living with HIV, and of those, two-thirds are in Sub-Saharan Africa and 1.3 million live in Zimbabwe [1]. It has an estimated national adult HIV prevalence of around 12.9% and continued new infections despite substantial gains in treatment coverage [2]. Dolutegravir (DTG) is the backbone of the preferred first-line antiretroviral (ARV) therapy (ART) for people living with HIV (PLWH) in most countries that have a non-nucleoside reverse transcriptase inhibitor (NNRTI) resistance of >10% [3]. DTG is an integrase strand transfer inhibitor with a high genetic barrier to resistance, favourable tolerability, and minimal drug–drug interactions [4,5]. In the current treatment regimen, it is administered as a once daily, single-tablet regimen of the low-cost generic fixed-dose of 300 mg tenofovir disoproxil fumarate, 300 mg lamivudine, and 50 mg dolutegravir (TLD) [3].

The use of ART has been a major improvement for HIV care, resulting in a life expectancy similar to that of the general population [6]. This has led to a shift from opportunistic infections to higher rates of non-communicable diseases (NCDs) [7]. DTG-based regimens have been linked to adverse interconnected clinical, metabolic, and biochemical abnormalities that share underlying mediators, mechanisms, and pathways [7–9]. The abnormalities that tend to cluster include abdominal obesity, hypertension, dyslipidemia, impaired glucose regulation and insulin resistance and these, if left untreated, collectively increase the risk of cardiovascular disease (CVD) and type 2 diabetes mellitus (T2DM) [9–14]. Weight gain associated with integrase inhibitors more broadly has been implicated as a pathway toward both impaired glycaemic control and dyslipidaemia [15]. The hypothesised mechanism of DTG hyperglycaemia is that DTG chelates intracellular magnesium, which is essential for glucose-stimulated insulin secretion, thereby contributing to impaired insulin secretion and glucose regulation [16].

Dyslipidemia refers to elevated triglycerides (TG) and low HDL-C, along with elevated apolipoprotein B (ApoB), small, dense LDL-C, and small HDL particles, all of which are independently atherogenic [17]. Several studies have reported dyslipidaemia in PLWH treated with DTG-based ART as compared to NNRTIs [8,18–20]. However, some studies have reported a modest to neutral effect on lipids for individuals on DTG-based ART compared to those on regimens with efavirenz [21,22].. In Zimbabwe specifically, data remain limited. A study by Zireva et al reported that PLWH who were co-infected with TB and receiving double-dose DTG as part of rifampicin-based TB treatment had a higher risk of hyperglycaemia and impaired glucose regulation [23]. A prospective cohort study at Parirenyatwa Hospital in Harare examined weight and lipid outcomes among adults 6 months after initiating DTG-based first-line ART [24].

As ART scale-up continues in Zimbabwe, generating data to monitor and mitigate the risk of cardiovascular disease and diabetes mellitus has become increasingly important, particularly in the context of an ageing population on ART. Given the substantial burden of hyperglycaemia and dyslipidaemia reported in comparable settings, local data are needed to inform and improve the quality of care. We therefore conducted an exploratory, hypothesis-generating cross-sectional study to estimate the burden of hyperglycaemia and dyslipidaemia, and to identify their correlates, among PLWH on first-line DTG-based ART.

## Methodology

### Study design and setting

This analytical cross-sectional study was conducted at the Parirenyatwa Hospital Opportunistic Infections Clinic in Harare, Zimbabwe. The clinic operates under Zimbabwe’s Ministry of Health and Child Care, under the Guidelines on HIV Prevention, Testing and Treatment of HIV in Zimbabwe of 2022 [3]. It serves as a tertiary referral site and also treats non-referral patients for routine drug refills and monitoring. The Clinic serves an urban primary catchment population of 1.2 to 1.5 million people in the greater Harare metropolitan area. However, as the apex healthcare institution, the tertiary population encompasses the entire nation of Zimbabwe (over 15 million people). The clinic provides comprehensive HIV management services, including pharmacy, laboratory, and clinical services. Blood samples for HbA1c and lipid profile are not routinely done at this clinic, but management and screening are guided by clinical presentation and medical history.

### Participant recruitment, data and sample collection

The study targeted adult PLWH (≥18 years) who had been on first–line DTG–based ART for at least 6 months. Consenting participants were recruited into the study during routine clinic visits from 20 January 2025–13 February 2025. Pregnant women and those who were acutely ill were excluded from the study. Trained research assistants administered a structured questionnaire to collect data from the participants and their medical records. The data collected included sociodemographic and clinical variables (ART initiation date, current ART regimen, viral load results, history of smoking, alcohol use, and level of physical activity). Physical activity, smoking status and alcohol use were each assessed using single self-reported binary (yes/no) items, without further specification of frequency, duration, intensity, or quantity ART adherence was self-reported by the study participants. Average household income was assessed as income from all the adults in the household.

A registered nurse measured blood pressure, weight and height. Height was measured in centimetres (cm) using a SECA stadiometer and weight in kilograms (kg) using a SECA digital scale. Body mass index (BMI) was calculated as: weight/height^2^. Blood pressure was measured with the participant in a seated position using the automated Omron Bronze upper arm blood pressure monitor BP510 [Omron Healthcare Inc.]. Two measurements were done 5 minutes apart, and the average reading was recorded.

A total of 15 mL of venous blood was collected, with 5 mL distributed into EDTA, sodium fluoride, and plain tubes, respectively. After collection, samples were transported to the laboratory in cooler boxes lined with ice packs. Specimens were centrifuged at 3000 rpm, and plasma and serum were extracted. Lipids, insulin and glucose were measured. EDTA whole blood was used to measure glycated haemoglobin (HbA1c). All samples were analysed within 4 hours of collection. Plasma insulin was used to calculate the Homeostatic Model Assessment of Insulin Resistance (HOMA-IR) [25].

### Laboratory processing

All analyses were conducted in accordance with the manufacturer’s protocols and the principles of good clinical laboratory practice. Serum lipids were quantified using combined enzymatic and colorimetric methods using the BIOELABS–200 Biobase (Biozone Co., Ltd., China) analyser. Insulin was assayed using a chemiluminescence immunoassay using the MAGLUMI-600 (Shenzhen New Industries Biomedical Engineering Co., Ltd (SNIBE)) and Biolumi Series analyser. HbA1c was measured in whole blood using nephelometry on the MISPA-I3 (Agape Diagnostics Ltd., India) analyser. Plasma glucose was measured using the glucose oxidase enzymatic method on the BIOELABS–200 Biobase (Biozone Co., Ltd., China) analyser.

### Variable definition and normal range

The main outcomes considered in this study were hyperglycaemia and dyslipidaemia. Hyperglycaemia was classified based on criteria recommended by the WHO, as glycated hemoglobin (HbA1c) ≥ 6.5%. Dyslipidaemia was defined as triglycerides ≥ 1.7 mmol/l, total cholesterol ≥5.2 mmol/l, HDL cholesterol < 1.03 mmol/l, LDL cholesterol ≥ 2.58 mmol/l or being on treatment according to the National Cholesterol Education Program Adult Treatment Panel III (NCEP ATP III). The independent variables were sociodemographic, clinical and anthropometric characteristics of the study participants. Obesity was defined based on the US Centers for Disease Control and Prevention criteria as a BMI ≥ 30 kg/m². Hypertension was classified as systolic BP ≥ 130 or diastolic BP ≥ 90 mmHg. Insulin resistance was defined as HOMA-IR ≥ 2.

### Ethical approval

The study received ethical approval from the Joint Research Ethics Committee (JREC) of Parirenyatwa Group of Hospitals and the University of Zimbabwe, Faculty of Medicine and Health Sciences (JREC ref: 133/2024) and the Medical Research Council of Zimbabwe (MRCZ) (MRCZ A/2519).

### Statistical analysis

Data were entered in REDCAP and exported to STATA, Texas, USA, version 18.5 for cleaning and analysis. Frequency and proportions were used to summarise the categorical variables such as marital status, education, and employment status, while the mean and standard deviation were used for normally distributed numerical data. Medians and interquartile ranges were utilised for data that was not normally distributed, such as age and household income. Categorical factors associated with hyperglycaemia and dyslipidaemia were analysed using Pearson Chi-square or Fischer’s exact tests. Student’s t-test or the Mann-Whitney equivalent was used for continuous variables. Univariate logistic regression was used to estimate odds ratios for each outcome (hyperglycaemia and dyslipidaemia) based on sociodemographic characteristics and clinical presentation. Additionally, binary and multivariate logistic regression models were fitted to analyse the complication outcomes in relation to sociodemographic characteristics and clinical factors. The significance level was kept at p < 0.05.

## Results

A total of 171 participants were enrolled in this study, of whom 67.8% were females, and the median age was 42.1 (32.4–50.2). Only 15 reported not having at least a secondary education. Nine were smokers, and 23% reported consuming alcohol. The majority of the participants (62.2%) reported not following an exercise program. Ninety-nine (57.9%) of the participants were either overweight or obese. Among the participants, 39.8% were hypertensive. (Tables 1 and 2).

**Table 1:** Sociodemographic, lifestyle and anthropometric characteristics of study participants, N = 171.

Variable	Frequency n (%)/ Median (Q1–Q3)
Age in years, median (Q1–Q3)	42.1 (32.4–50.2)
**Sex**	
Male	55 (32.2)
Female	116 (67.8)
**Marital status**	
Single	41 (24.0)
Married	88 (51.5)
Divorced	21 (12.3)
Widowed	21 (12.3)
**Education level**	
Primary	15 (8.8)
Secondary	123 (71.9)
Tertiary	33 (19.3)
**Employment status**	
Unemployed	55 (32.2)
Self-employed	64 (37.4)
Formally employed	52 (30.4)
**Average monthly household income in USD, median (Q1–Q3)**	200 (60–300)
**Smoker**	
No	162 (94.7)
Yes	9 (5.3)
**Consumes alcohol**	
No	131 (76.6)
Yes	40 (23.4)
**Ever used illicit drugs**	
No	165 (96.5)
Yes	6 (3.5)
**Follows an exercise program**	
No	108 (63.2)
Yes	63 (36.8)
**Manual work for at least 30 minutes per week**	
Never	31 (18.1)
Once	20 (11.7)
Twice	29 (17.0)
Three times	20 (11.7)
More than three times	71 (41.5)
**BMI (kg/m²**)	
Severely underweight (<15)	1 (0.6)
Underweight (15–18.5)	10 (5.9)
Normal (18.6–24.9)	61 (35.7)
Overweight (25–29.9)	49 (28.7)
Obese (30+)	50 (29.2)
**Systolic blood pressure (mmHg), median (Q1–Q3)**	131.0 (119.0–145.0)
**Diastolic blood pressure (mmHg), median (Q1–Q3)**	84.0 (74.0–92.0)
**Blood pressure**	
Hypotensive (<90/ < 60)	1 (0.6)
Normotensive (<140 & ≥ 90/ < 90 & ≥ 60)	102 (59.7)
Hypertensive (≥140/ ≥ 90)	68 (39.8)

BMI, body mass index; Q1–Q3, first to third interquartile range*.*

**Table 2:** Laboratory characteristics of study participants, N = 171. There were 23 (13.4%) participants with hyperglycaemia, and 56.1% had insulin resistance. The number who had hypercholesterolaemia was 20 (10.3%), those with non-ideal HDL-C 98 (50.5), raised LDL-C 64 (33.3%) and raised Triglycerides 31 (16%).

Variable	Frequency n (%)/ Median (Q1–Q3)
HbA1c (%), median (Q1–Q3)	5.8 (5.5–6.1)
**HbA1c**	
Normoglycaemia (4.0– < 5.7%)	80 (46.8)
Impaired glucose regulation (5.7– < 6.5%)	68 (39.8)
Hyperglycaemia (≥6.5%)	23 (13.4)
**HOMA-IR**	
≤ 2	75 (43.9)
> 2	96 (56.1)
Total cholesterol (mmol/L), median (Q1–Q3)	3.8 (3.2–4.7)
**Total cholesterol**	
Ideal level (<5.2 mmol/L)	174 (89.7)
Hypercholesterolaemia (≥5.2 mmol/L)	20 (10.3)
HDL-C (mmol/L), median (Q1–Q3)	1.2 (1.0–1.4)
**HDL-C**	
Ideal level (>1.2 mmol/L)	96 (49.5)
Non-ideal level (≤1.2 mmol/L)	98 (50.5)
LDL-C (mmol/L), median (Q1–Q3)	2.2 (1.7–2.9)
**LDL-C**	
Ideal level (<2.58 mmol/L)	128 (66.7)
Non-ideal level (≥2.58 mmol/L)	64 (33.3)
Triglycerides (mmol/L), median (Q1–Q3)	0.96 (0.69–1.36)
**Triglycerides category**	
Ideal level (<1.7 mmol/L)	163 (84.0)
Hypertriglyceridaemia (≥1.7 mmol/L)	31 (16.0)

HbA1c, glycated haemoglobin; HDL-C, high-density lipoprotein cholesterol; HOMA-IR, homeostatic model assessment of insulin resistance; LDL-C, low-density lipoprotein cholesterol; Q1–Q3, first to third interquartile range.

The prevalences of dyslipidaemia and hyperglycaemia were 74.3% and 13.4%, respectively (Table 3).

**Table 3:** Prevalence of hyperglycaemia and dyslipidaemia.

Variable	Prevalence n (%)
**Hyperglycaemia**	
No	148 (86.6)
Yes	23 (13.4)
**Dyslipidaemia**	
No	44 (25.7)
Yes	127 (74.3)

In univariate analysis, age was associated with hyperglycaemia. In multivariate explanatory logistic regression, increasing age was the only variable found to have a positive association with hyperglycaemia, adjusted odds ratio (95% confidence interval), 1.08 (1.03–1.13), p = 0.002. (Table 4)

**Table 4:** Factors associated with hyperglycaemia N = 171.

Variable	Hyperglycaemia N = 23 (13.4%)	No hyperglycaemia N = 148 (86.6%)	Crude OR (95% CI)	P-value	Adjusted OR (95% CI)	P-value
**Age in years, median (Q1–Q3)**	52 (46–56)	41 (32–48)	1.10 (1.05–1.16)	<0.001	1.09 (1.03–1.15)	0.002
**Sex**						
Female	14 (12.1)	102 (87.9)	Ref		Ref	
Male	9 (16.4)	46 (83.6)	1.43 (0.58–3.53)	0.444	1.79 (0.47–6.81)	0.395
**BMI, median (Q1–Q3)**	28.1 (22.9–33.6)	26.0 (22.0–30.5)	1.06 (1.00–1.13)	0.064	1.06 (0.98–1.15)	0.156
**Elevated blood pressure**						
No	11 (10.7)	92 (89.3)	Ref		Ref	
Yes	12 (17.6)	56 (82.4)	1.79 (0.74–4.33)	0.195	0.90 (0.32–2.55)	0.844
**Education level**						
Primary	3 (20.0)	12 (80.0)	Ref		Ref	
Secondary	14 (11.4)	109 (86.6)	0.51 (0.13–2.05)	0.345	0.57 (0.12–2.65)	0.478
Tertiary	6 (18.2)	27 (81.8)	0.89 (0.19–4.16)	0.881	0.98 (0.15–5.81)	0.936
**Employment status**						
Unemployed	6 (10.9)	49 (89.1)	Ref		Ref	
Self-employed	11 (17.2)	31 (82.8)	1.69 (0.58–4.93)	0.333	0.90 (0.26–3.15)	0.873
Formally employed	6 (11.5)	46 (88.5)	1.07 (0.32–3.54)	0.918	0.62 (0.15–2.65)	0.526
**Average monthly household income in USD, median (Q1–Q3)**	150 (80–250)	200 (60–300)	1.00 (0.99–1.00)	0.469	0.99 (0.99–1.00)	0.934
**Smoker**						
No	23 (15.4)	137 (84.6)	Ref		Ref	
Yes	1 (11.1)	8 (88.9)	0.69 (0.08–5.72)	0.727	0.69 (0.06–7.38)	0.758
**Consumes alcohol**						
No	15 (13.7)	113 (86.3)	Ref		Ref	
Yes	5 (12.5)	34 (87.5)	0.90 (0.31–2.59)	0.841	0.86 (0.24–3.17)	0.838
**Follows an exercise program**						
No	14 (13.0)	94 (87.0)	Ref		Ref	
Yes	9 (14.3)	54 (85.7)	1.12 (0.45–2.76)	0.807	1.08 (0.37–3.15)	0.894
**ART duration (years), median (Q1–Q3)**	5.1 (3.1–6.0)	4.1 (1.6–6.1)	1.09 (0.92–1.29)	0.323	1.07 (0.85–1.34)	0.582
**Viral load, median (Q1–Q3)**	0 (0–25)	0 (0–20)	0.99 (0.99–1.00)	0.727	0.99 (0.99–1.00)	0.815

ART, antiretroviral therapy; BMI, body mass index; CI, confidence interval; OR, odds ratio; Q1–Q3, first to third interquartile range; Ref, reference category. Adjusted odds ratios were derived from a multivariable logistic regression model.

In univariate analysis age, employment status and elevated blood pressure were significantly associated with dyslipidaemia. In the multivariate explanatory logistic regression model, we found a positive association between elevated blood pressure and dyslipidaemia; adjusted odds ratio (95% confidence interval), 2.64 (1.03–6.78), p = 0.043 (Table 5).

**Table 5:** Factors associated with dyslipidaemia N = 171.

Variable	Dyslipidaemia N = 127 (74.3%)	No dyslipidaemia N = 44 (25.7%)	Crude OR (95% CI)	P-value	Adjusted OR (95% CI)	P-value
**Age in years, median (Q1–Q3)**	45 (36–51)	38 (29–44)	1.05 (1.02–1.09)	0.001	1.03 (0.99–1.07)	0.109
**Sex**						
Female	82 (70.7)	34 (29.3)	Ref		Ref	
Male	45 (81.8)	10 (18.2)	1.87 (0.84–4.12)	0.123	2.92 (0.94–9.07)	0.063
**BMI, median (Q1–Q3)**	27.7 (22.9–30.8)	25.1 (21.2–31.2)	1.03 (0.98–1.09)	0.233	1.02 (0.95–1.10)	0.509
**Elevated blood pressure**						
No	67 (65.1)	36 (35.0)	Ref		Ref	
Yes	60 (88.2)	8 (11.8)	4.03 (1.74–9.35)	0.001	2.64 (1.03–6.78)	0.043
**Education level**						
Primary	13 (86.7)	2 (13.3)	Ref		Ref	
Secondary	86 (69.9)	37 (30.1)	0.36 (0.08–1.66)	0.190	0.33 (0.06–1.84)	0.208
Tertiary	28 (84.9)	5 (15.1)	0.86 (0.15–5.04)	0.869	0.64 (0.08–5.00)	0.670
**Employment status**						
Unemployed	34 (61.8)	21 (38.2)	Ref		Ref	
Self-employed	47 (73.4)	17 (26.8)	1.71 (0.79–3.71)	0.177	1.36 (0.53–3.51)	0.526
Formally employed	46 (88.5)	6 (11.5)	4.74 (1.73–13.00)	0.003	3.20 (0.94–10.87)	0.063
**Average monthly household income in USD, median (Q1–Q3)**	200 (60–300)	150 (60–200)	1.00 (0.99–1.00)	0.092	1.00 (1.00–1.00)	0.641
**Smoker**						
No	121 (74.7)	41 (25.3)	Ref		Ref	
Yes	6 (66.7)	3 (33.3)	0.68 (0.16–2.83)	0.594	0.37 (0.06–2.28)	0.285
**Consumes alcohol**						
No	98 (74.8)	33 (25.2)	Ref		Ref	
Yes	29 (72.5)	11 (27.5)	0.89 (0.40–1.97)	0.770	0.56 (0.21–1.53)	0.262
**Follows an exercise program**						
No	82 (75.9)	26 (24.1)	Ref		Ref	
Yes	45 (74.1)	18 (28.6)	0.79 (0.39–1.60)	0.517	0.62 (0.27–1.42)	0.261
**ART duration (years), median (Q1–Q3)**	4.1 (1.6–6.1)	4.1 (2.1–6.1)	0.96 (0.84–1.10)	0.567	0.95 (0.81–1.11)	0.519
**Viral load, median (Q1–Q3)**	0 (0–21)	0 (0–20)	1.00 (0.99–1.00)	0.735	1.00 (1.00–1.00)	0.577

ART, antiretroviral therapy; BMI, body mass index; CI, confidence interval; OR, odds ratio; Q1–Q3, first to third interquartile range; Ref, reference category. Adjusted odds ratios were derived from a multivariable logistic regression model.

In univariate analysis, age was associated with hyperglycaemia. In multivariate explanatory logistic regression increasing age was the only variable found to have a positive association with hyperglycaemia, adjusted odds ratio (95% confidence interval), 1.08 (1.03–1.13), p = 0.002. (Table 4)

In univariate analysis age, employment status and elevated blood pressure were significantly associated with dyslipidaemia. In multivariate explanatory logistic regression model, we found a positive association between elevated blood pressure and dyslipidaemia; adjusted odds ratio (95% confidence interval), 2.64 (1.03–6.78), p = 0.043, (Table 5).

## Discussion

This study set out to investigate the burden of hyperglycaemia and dyslipidaemia among Zimbabwean adults living with HIV who are on DTG-based first-line ART. It highlights the prevalence, patterns and correlates of these two metabolic complications in this population. The introduction of DTG has been a significant advancement in HIV management, yet it raises concerns regarding the associated metabolic side effects [8,19,26–28]. In this study, 116 (67.8%) were females. This may reflect the demographic distribution of individuals accessing HIV care in this setting. According to the UNAIDS report of 2024, the treatment coverage for men is still lagging [29].

Glycaemic indicators in this study indicated that 39.8% had poor glycaemic control and 13.4% met hyperglycaemia criteria for diabetes. This prevalence aligns with growing evidence that PLWH are at increased risk of developing type 2 diabetes mellitus. In Zimbabwe, diabetes affects an estimated 1.5% to 4.6% of the adult population [30]. In a study that compared hyperglycaemia in PLWH on DTG vs efavirenz, Jemal *et al* reported an overall prevalence of 13.2%, with 17.2% for those on DTG and 9.2% for the efavirenz arm [8]. We found an almost similar prevalence of hyperglycaemia to that reported in the same population in Uganda (12.8%) [31] whereas a study in Southern Ethiopia reported a much higher prevalence of 24.4% [32]. A study in Iran reported an even higher prevalence of 55.6% [33]. The observed variations in prevalence could be attributed to several factors. For instance, variations in the ART exposure history among participants, differences in blood glucose testing methods and differences in sample size. In our study, we included participants who had been on DTG for at least 6 months, whereas the study in Southern Ethiopia (prevalence 24.4%) included participants who had transitioned to DTG-based ART. Genetic differences may also play a part, hence the high prevalence reported in Iran (55.6%).

The findings of hyperglycaemia in this study support earlier reports from case studies and controlled clinical trials. Severe or life-threatening hyperglycaemia was documented in case reports by Hailu et al, and Lamonde et al [34,35]. In some of the reported cases, hyperglycaemia was resolved after discontinuation of DTG [35]. The ADVANCE and NAMSAL trials reported that raised fasting glucose was associated with DTG use, likely driven by obesity [36]. In contrast, data collected from the Dat’AIDS cohort study in France showed that INSTI-based regimens were not significantly associated with incident diabetes compared with regimens containing an NNRTI or a PI [37]. A study in Arizona, United States reported that DTG may cause a moderate increase in plasma sugar after three to six months of therapy that warrants escalation of therapy [38].

In univariate analysis, age was significantly associated with hyperglycaemia (crude odds ratio (COR): 1.09, 95% CI: 1.05–1.15, p < 0.001) and again in multivariate explanatory logistic regression, increasing age was the only variable found to have a positive association with hyperglycaemia, adjusted odds ratio (95% confidence interval): 1.08 (1.03–1.13), p = 0.0021. The crude odds ratio (COR) of 1.09 indicates that each one-year increase in age was associated with a 9% increase in the odds of hyperglycaemia before adjusting for other covariates. Several studies have reported that age is a determinant of hyperglycaemia in PLWH on DTG-based ART [32,39–41]. Ageing is a well-established risk factor for impaired glucose metabolism and type 2 diabetes due to progressive physiological changes that reduce glucose homeostasis [42]. With age, the pancreatic β-cell function declines, reducing the capacity for insulin secretion in response to glucose [43]. As age is a non-modifiable risk factor, these findings underscore the need for targeted glycaemic monitoring among older adults receiving DTG-based ART and as the population is ageing with HIV.

We did not find any association between BMI and hyperglycaemia in this study, although several studies have observed an association {Citation}Jemal et al (in a Ugandan study) reported that for a kg/m2 increase in BMI, people taking DTG were 66% more likely to have hyperglycaemia [8]. Excess adiposity as seen in obesity promotes insulin resistance through chronic low-grade inflammation, altered adipokine secretion, and ectopic lipid accumulation, leading to hyperglycaemia [44]. The absence of an association between BMI and hyperglycaemia may reflect the limitations of BMI as a surrogate measure of adiposity. BMI does not assess visceral fat accumulation, which is more metabolically active and has a stronger relationship with insulin resistance and impaired glucose metabolism [45].

Clinically, the observed prevalence of hyperglycaemia is significant because sustained hyperglycaemia is known to accelerate the development of both macrovascular and microvascular complications [46] thereby increasing morbidity and mortality in this population.

Among the study participants, 74.3% had at least one abnormality in the lipid profile. The most affected component was HDL-C, with hypoalphalipoproteinemia present in 50.5% of participants, followed by elevated LDL cholesterol (33.3%). Hypertriglyceridemia and hypercholesterolemia were less common, at 16% and 10.3%, respectively. The prevalence result is consistent with findings from Uganda (79.7%), Ethiopia (74%), Rwanda (82.14%), and Tanzania (77.5%), but higher than the reported prevalence in Central Uganda (44%), South-west Ethiopia, and Shewa, Ethiopia (59% and 44.4%). The observed variations may be explained by factors including differences in the effects of ART regimens on lipid metabolism, the duration of ART exposure, and consequent alterations in lipid profiles. It is also unclear how the prevalence and among our study participants compares with that in the general population.

In this study, low HDL-C affected just over half of participants (50.5%), while elevated LDL-C affected roughly one-third (33.3%). Hypercholesterolemia (10.3%) and hypertriglyceridemia (16.0%) were comparatively uncommon. This lipid subfraction pattern where low HDL-C is the leading abnormality and total cholesterol and triglycerides remain largely within ideal ranges is consistent with observations in [20, 47]. The consistency of low HDL-C as the predominant abnormality across these settings despite differences in population and geography suggests a shared underlying mechanism. This mechanism is related to DTG’s association with weight gain and the resulting insulin resistance, which is known to suppress HDL-C synthesis and promote HDL particle catabolism [48]. However, a cross-sectional study of 382 HIV/AIDS patients in two national referral hospitals in Asmara, Eritrea found that among individual lipid markers, the order of prevalence was LDL-C (55.8%), low HDL-C (44%), triglycerides (33.2%), and high total cholesterol (29.1%). Overall dyslipidemia prevalence was 86.6%, with increased LDL-C (213 patients, 55.8%) being the predominant [49]. Chakanetsa et al. in a Zimbabwean found DTG initiation was associated with an improvement in lipid profile (falling dyslipidaemia prevalence, rising HDL-C, falling triglycerides) over 6 months of follow-up [24]. Whereas our study found a 74.3% dyslipidaemia burden**,** with low HDL-C still the dominant abnormality. This difference may be explained by our cross-sectional study having longer average DTG exposure, where early lipid improvements may reverse over time,

Older age was associated with higher odds of dyslipidaemia in unadjusted analysis (crude OR 1.05 per year, 95% CI 1.02–1.09, p = 0.001), consistent with age-related deterioration in lipid metabolism. However, this association was attenuated and lost statistical significance after adjustment (adjusted OR 1.03, 95% CI 0.99–1.07, p = 0.109), suggesting that age may act substantially through its correlation with other factors in the model, plausibly blood pressure or employment-related socioeconomic status rather than as an independent driver of dyslipidaemia risk in this population. Employment status showed a similar pattern. Formally employed participants had substantially higher odds of dyslipidaemia than unemployed participants in unadjusted analysis (crude OR 4.74, 95% CI 1.73–13.00, p = 0.003), but this association weakened after adjustment and did not reach conventional statistical significance (adjusted OR 3.20, 95% CI 0.94–10.87, p = 0.063). Though the point estimate remained substantial and the confidence interval suggests the study may have been underpowered to confirm this association definitively. This may be explained by the already known associations between higher socioeconomic status, more sedentary occupational patterns, and dietary transitions toward low-protein, high-calorie foods in urban African settings including Zimbabwe [50,51]. This would be expected to elevate dyslipidaemia risk independent of age. Sex showed a similar borderline pattern, with male participants trending toward higher adjusted odds of dyslipidaemia (adjusted OR 2.92, 95% CI 0.94–9.07, p = 0.063). This finding should be treated as hypothesis-generating rather than conclusive.

Elevated blood pressure emerged as the strongest independent correlate of dyslipidaemia, with participants who had elevated blood pressure having over twice the odds of dyslipidaemia after adjustment for other covariates (adjusted OR 2.64, 95% CI 1.03–6.78, p = 0.043). Though the confidence interval (crossing from barely significant to nearly 7-fold) is wide, the direction and magnitude are broadly consistent with what’s emerging in the DTG literature. A southern Ethiopian study found that initiating or switching to DTG-based regimens was significantly associated with high blood pressure compared to efavirenz-based regimens [52]. Separately, a Ghanaian cohort found that over half of adults with HIV initiated on a DTG-based regimen developed hypertension, with a quarter developing it within 6 months of starting DTG [53]. Multiple mechanisms including adipogenesis, activation of adipocytes, the renin-angiotensin system, immune reconstitution, chronic systemic and vascular inflammation, and renal insufficiency can all impact the development of hypertension in PLWH [52]. DTG-associated weight gain doesn’t act on lipids or blood pressure separately. This suggests but is not conclusive that DTG itself (not just incidental aging or unrelated comorbidity) might be contributing to the elevated blood pressure seen alongside dyslipidaemia.

Notably, several variables commonly associated with dyslipidaemia in similar studies such as BMI, smoking status, alcohol consumption, and exercise participation showed no significant association in either crude or adjusted analyses. This may reflect genuine absence of association in this specific population. This could equally reflect measurement limitations, such as self-reported smoking, alcohol use, and exercise, which are prone to social desirability bias. ART duration and viral load which are known proxies for treatment stability and immune recovery [54] were similarly not statistically significantly associated with dyslipidaemia. While our study enrolled participants who had been on DTG-based ART for varying durations, the cross-sectional design does not allow us to conclude the true long-term effects of DTG on lipid profiles. Longitudinal studies with baseline pre-ART measurements or studies with a comparator group are needed to establish this more definitively.

The high prevalence of dyslipidaemia is associated with significant health problems. Elevated LDL cholesterol and triglycerides, combined with low HDL cholesterol, promote atherosclerosis and increase the risk of cardiovascular events. Excess body fat, especially visceral fat, is linked to higher rates of hypertension and CVD [23,25,39]. Lipid profiles and related abnormalities result from a multifactorial interplay involving immunological alterations, hormonal regulation, and genetic predisposition. Our study enrolled participants who had been on DTG for varying lengths of time and could indicate the long-term effects of DTG on lipid profiles.

We would like to acknowledge the limitations of this study. The cross-sectional design precludes causal or temporal inference between DTG exposure and the observed hyperglycaemia and dyslipidaemia. Future studies may include a non-DTG comparator group or a follow-up study to include baseline data. Potential confounders such as dietary intake, physical activity, and alcohol use were self-reported and may be subject to recall and social desirability bias, raising the possibility of residual confounding in the determinants analysis. The study may have missed PLWH not engaged in routine care who may carry a different risk profile. Despite these limitations, the findings provide important, setting-specific evidence to inform cardiometabolic screening among Zimbabwean adults on DTG-based first-line ART. HbA1c and lipid values were measured directly using validated laboratory assays, reducing misclassification bias compared to studies relying on medical record review alone. Examining both hyperglycaemia and dyslipidaemia together (rather than one in isolation) captures a better cardiometabolic picture given how tightly these conditions cluster mechanistically.

## Conclusion

Our study reveals a significant prevalence of dyslipidaemia and hyperglycaemia among individuals on DTG-based ART, with age emerging as a key correlate of this cardiometabolic burden. As the population of PLWH continues to age on long-term ART, the cumulative risk of metabolic and cardiovascular complications is increasing. This highlights the need for routine, integrated screening within HIV care programmes. Coordinated management of these comorbidities, alongside strengthened monitoring and evaluation systems, will be essential to mitigating long-term cardiovascular risk and sustaining the success of DTG-based ART in Zimbabwe.

## Supporting information

S1 Data Dataset. (XLSX)
